# 

**Published:** 1914-01-31

**Authors:** 


### Gifts and Bequests.

The Lord Mayor has sent to Dr. Barnardo's Homes 25 guineas from the Association of Lancastrians in London.

Miss Emma Augusta Charlotte Cust, of West Cowes, has left £2,000 to the British Home for Incurables at Streatham.

The Middlesex Hospital Cancer Charity has received from the executors of the late Mr. Rutherford £500 for cancer research.

The Middlesex Hospital Cancer Charity has received from Miss Fanny Marriage, as the result of her sale of jam and pickles, a further £103.

The Late Mr. W. H. Stevens, J.P., has left to the Leicester Royal Infirmary Children's Hospital £200, payable on the death of Mrs. Stevens.

King Edward's Hospital Fund for London has received at the Bank of England from the League of Mercy its contribution for the year 1913 of £14,000.

Miss Amanda Hinchliffe, of Southport, has left £4,000 to the Royal Halifax Infirmary, £1,000 to the Bath Hospital at Harrogate, and £500 to the North of England Children's Sanatorium.

### Appointments.

DR. J. F. GWYNNE has resigned his appointment as second assistant medical officer at Southwark Infirmary.

MR. HAROLD L. WHALE, M.D., B.C., B.A. (Cantab.), F.R.C.S. (Eng.), has been appointed surgeon to the Throat, Nose, and Ear Department of the Hampstead and North-West London Hospital.

MR. HAROLD WIGG has been appointed secretary of the recently opened Empire Hospital for Paying Patients, Vincent Square. Mr. Wigg was formerly secretary to the Walsall and District Hospital till he came to London to take up another appointment last year.

DR. RICHARD A. BANBURY, third assistant medical officer at Southwark Infirmary, has been promoted to the position of second assistant medical officer, with remuneration at the rate of £120 per annum, rising by £10 at Midsummer next to a maximum of £140 per annum at Midsummer 1915, with the usual emoluments.

DR. A. MIDDLETON HEWAT, M.D., Ch.B., D.P.H., has been appointed tuberculosis officer and assistant medical officer of health to Preston. Dr. Hewat gained in 1909 a research scholarship in tuberculosis at the Royal Victoria Hospital for Consumption, Edinburgh, became resident physician there, and studied anti-tuberculosis work under Sir Robert Philip. His previous post has been deputy county medical officer of health and tuberculosis officer for East Suffolk.

THE London County Council Mental Hospitals Committee have accepted the resignation of Dr. J. P. Candler, assistant pathologist at the Pathological Laboratory, Claybury, who has been appointed a medical inspector under the Local Government Board. They have arranged for the *post-mortem* work hitherto performed by him at the Pathological Laboratory to be carried out for the present by one of the assistant medical officers at Claybury Mental Hospital.

RATES OF SUBSCRIPTION: Twelvemonths, 6/6; Six months, 4/-; Three months, 2/-, post free to any address in the United Kingdom. Abroad, Twelvemonths, 8/8; Six months, 5/-; Three months, 2/6, post free.—Address The Subscription Dept., "THE HOSPITAL," The Hospital Building, 23/29 Southampton Street, Strand, London, W.C.

